# Advancements in Complementary Metal-Oxide Semiconductor-Compatible Tunnel Barrier Engineered Charge-Trapping Synaptic Transistors for Bio-Inspired Neural Networks in Harsh Environments

**DOI:** 10.3390/biomimetics8060506

**Published:** 2023-10-23

**Authors:** Dong-Hee Lee, Hamin Park, Won-Ju Cho

**Affiliations:** 1 Department of Electronic Materials Engineering, Kwangwoon University, Gwangun-ro 20, Nowon-gu, Seoul 01897, Republic of Korea; zpsxlzje@naver.com; 2 Department of Electronic Engineering, Kwangwoon University, Gwangun-ro 20, Nowon-gu, Seoul 01897, Republic of Korea

**Keywords:** charge trapping/de-trapping, conductance modulation, silicon-on-insulator (SOI), synaptic transistor, high-*k* dielectrics, high-temperature operation, artificial neural networks

## Abstract

This study aimed to propose a silicon-on-insulator (SOI)-based charge-trapping synaptic transistor with engineered tunnel barriers using high-*k* dielectrics for artificial synapse electronics capable of operating at high temperatures. The transistor employed sequential electron trapping and de-trapping in the charge storage medium, facilitating gradual modulation of the silicon channel conductance. The engineered tunnel barrier structure (SiO_2_/Si_3_N_4_/SiO_2_), coupled with the high-*k* charge-trapping layer of HfO_2_ and high-k blocking layer of Al_2_O_3_, enabled reliable long-term potentiation/depression behaviors within a short gate stimulus time (100 μs), even under elevated temperatures (75 and 125 °C). Conductance variability was determined by the number of gate stimuli reflected in the maximum excitatory postsynaptic current (EPSC) and the residual EPSC ratio. Moreover, we analyzed the Arrhenius relationship between the EPSC as a function of the gate pulse number (N = 1–100) and the measured temperatures (25, 75, and 125 °C), allowing us to deduce the charge trap activation energy. A learning simulation was performed to assess the pattern recognition capabilities of the neuromorphic computing system using the modified National Institute of Standards and Technology datasheets. This study demonstrates high-reliability silicon channel conductance modulation and proposes in-memory computing capabilities for artificial neural networks using SOI-based charge-trapping synaptic transistors.

## 1. Introduction

Synaptic devices have garnered considerable attention as potential replacements for conventional von Neumann computing architectures and Boolean logic [1,2]. Inspired by biological neurons and synapses in the human brain, synaptic devices are primarily used for artificial intelligence (AI) processing applications [3,4]. The fundamental roles of electrical synapses in brain-mimicking computing systems include massive parallelism, high operating speeds, low power consumption, and efficient storage/computation interconnection. Such systems demonstrate the capability of solving a wide range of problems in inference, recognition, optimization, and control [5,6].

Recently, diverse nonvolatile memory devices with various device structures and materials have been proposed for electronic synapses. In particular, two-terminal devices, such as phase-change and spintronic devices and memristors, have gained widespread attention owing to their potential for high integration in cross-array structures. However, these devices are susceptible to sneaking current issues and disturbances caused by excessive noise, necessitating additional control elements because of the structural connection between the stimulus and output domains [7,8]. In contrast, multiterminal synaptic transistors offer better suitability for complex neural computing functions and real-time processing owing to their independence from the stimulus/output domains. Multiterminal devices with various memory elements, such as floating-, electrolyte-, and ferroelectric-gate transistors, to implement synaptic behavior have been widely reported [9,10,11].

Meanwhile, the floating-gate device, a fundamental unit cell in modern flash memory, is rapidly approaching its intrinsic limits owing to its scaling down. Reducing the tunnel oxide thickness leads to inherent limitations in the scaling factors associated with floating-gate coupling, where the state of the adjacent cells is coupled via the gate leakage current and gate capacitance [12,13]. To address these challenges, charge trap flash (CTF) memory with advantageous structural attributes has emerged as an attractive candidate to ensure continuity in vertical scaling and overcome gate coupling limitations in conventional flash memory [14,15]. However, the SiO_2_/Si_3_N_4_/SiO_2_ structure in silicon–oxide–nitride–silicon (SONOS) presents fundamental issues in balancing data retention and erase speed. To enhance write and erase operations in SONOS memory, reducing the tunnel oxide thickness is essential [16,17,18]. On the other hand, maintaining a constant tunnel oxide thickness is crucial for long data retention to minimize leakage current problems caused by stress. To overcome the limitations of CTF-type nonvolatile memory, the use of high-*k* materials, such as zirconium, hafnium, tantalum, and aluminum oxides, in the tunneling and charge-trapping layers (CTLs) has been proposed to improve performance. These high-*k* materials exhibit relatively larger conduction band offsets with Si and higher dielectric constants and trap densities compared with conventional materials, resulting in superior charge-trapping characteristics [19,20,21].

In this study, we proposed a silicon-on-insulator (SOI)-based charge-trapping synaptic transistor with tunnel barrier engineering that utilizes high-*k* materials. The active application of charge trapping in nonvolatile memory devices has great potential for neural applications, offering high reliability, stable endurance, good data retention, and high fault tolerance [9]. Furthermore, its superior complementary metal-oxide semiconductor (CMOS) process compatibility enables ultrahigh device integration, making it a promising candidate for future artificial synaptic devices. We systematically evaluated the fundamental electrical properties and artificial neural behavior of the SOI-based charge-trapping synaptic transistors. Tunnel barrier engineering to enhance carrier injection by reducing the effective tunneling thickness is achieved via the ONO structure, where SiO_2_/Si_3_N_4_/SiO_2_ is laminated with a thin layer, and the HfO_2_ CTL and Al_2_O_3_ blocking layer (BL) are sequentially stacked to implement the charge-trapping synaptic transistor. By utilizing gate pulse stimuli, electrons are effectively trapped and de-trapped in a charge storage medium to mimic highly reliable synaptic action. The silicon channel conductance is gradually modulated via consecutive gate stimuli, and individual states are maintained for extended periods. This conductance variability is determined by the maximum excitatory-postsynaptic current (EPSC) and residual EPSC ratios, demonstrating a transition from short-term memory (STM) to long-term memory (LTM) mechanisms. We also evaluated the Arrhenius relationship between the EPSC and the number of gate pulse stimulations at measured temperatures (25, 75, and 125 °C) to gain insight into the trap activation energy (*E_a_*). Moreover, the silicon channel conductance was successfully potentiated and depressed by short gate pulse stimuli (100 μs) during repeated endurance cycles at room temperature (25 °C) and even at high temperatures (75 and 125 °C). This highly reliable and discriminable conductance variability highlights the potential of SOI-based charge-trapping synaptic transistors for artificial neural network (ANN) systems. To demonstrate the performance of the proposed CMOS-compatible charge-trapping synaptic transistor in neuromorphic systems, we conducted recognition simulations using a modified National Institute of Standards and Technology (MNIST) dataset via a multilayer ANN model. The results exhibited high learning accuracy even at high temperatures, underscoring its potential as a viable component for neuromorphic computing and AI applications in harsh environments.

## 2. Materials and Methods

### 2.1. Fabrication of a Silicon-on-Insulator-Based Charge-Trapping Synaptic Transistor with Engineered Tunnel Barriers

The fabrication process for the charge-trapping synaptic transistors involves the utilization of (100) oriented p-type bonded and etch-back SOI wafers as the initial materials, followed by a standard Radio Corporation of America cleaning process to eliminate particles. The top silicon layer, with a doping concentration of 1 × 10^15^ cm^−3^, has a thickness of 100 nm, whereas the buried oxide layer measures 750 nm in thickness. The active regions of the top silicon undergo patterning using photolithography and are formed via CF_4_ reactive ion etching (RIE). Subsequently, a 100 nm thick in situ phosphorus-doped polysilicon layer is deposited for the source/drain (S/D) by employing a low-pressure chemical vapor deposition (LPCVD) process at 650 °C. Post-deposition annealing is performed via rapid thermal annealing at 850 °C for 30 s in an N_2_ ambient atmosphere. A 40 nm thick silicon channel layer with a low S/D series resistance is then achieved by RIE, which involves removing the n^+^-doped poly-Si layer (except for the S/D regions) and thinning the top silicon layer. Any surface damage and roughness on the silicon channel resulting from the thinning process are meticulously eliminated by treatment with an ammonia peroxide mixture solution. The channels are defined to have dimensions of 10 μm width and 10 μm length. The subsequent critical elements, engineered tunnel barriers CTL and BL, are established in the following sequence: A stack of ONO structures is deposited onto the silicon channel with layer thicknesses of 2 nm (SiO_2_, thermal oxidation), 2 nm (Si_3_N_4_, LPCVD), and 3 nm (SiO_2_, LPCVD), forming a variable oxide thickness (VARIOT) tunnel barrier. Subsequently, a 5 nm thick HfO_2_ film for the CTL and a 12 nm thick Al_2_O_3_ film for the BL is deposited using atomic layer deposition. The final step involves forming gas annealing at 450 °C in a 2% H_2_/N_2_ mixture at ambient temperature for 30 min following the deposition of a 20/100 nm thick Ni/Al gate electrode using an electron beam evaporator.

### 2.2. Characterizations

To ensure precise measurements and mitigate the effects of electrical noise and external light, CMOS-compatible charge-trapping synaptic transistors were placed within a shielded dark box on a probe station. The electrical characteristics of the proposed transistors were measured using an Agilent 4156 B Precision Semiconductor Parameter Analyzer (Hewlett-Packard Co., Palo Alto, CA, USA). Synaptic modulation was assessed by applying electrical pulse stimulation generated using an Agilent 8110A pulse generator (Hewlett-Packard Co.).

## 3. Results and Discussion

### 3.1. Electrical Characteristics of Complementary Metal-Oxide Semiconductor-Compatible Charge-Trapping Synaptic Transistors

**Figure 1:**
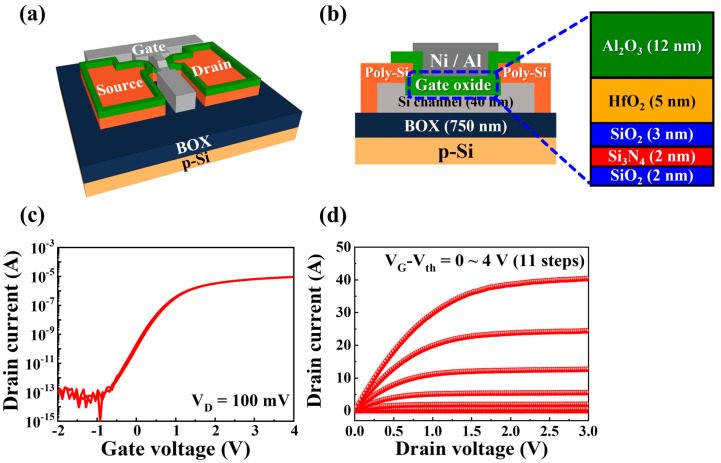
(**a**) Three-dimensional schematic of silicon-on-insulator (SOI)-based charge-trapping synaptic transistors with SiO_2_/Si_3_N_4_/SiO_2_-engineered tunnel barriers. (**b**) Vertical cross-section of gate metal/Al_2_O_3_/HfO_2_/SiO_2_/Si_3_N_4_/SiO_2_/Si (MAHONOS) stack. Typical electrical characteristics of (**c**) transfer (I_D_-V_G_) and (**d**) output (I_D_-V_D_) curves.

Figure 1a,b present the schematics of the three-dimensional and vertical cross-sectional structures of the fabricated SOI-based charge-trapping synaptic transistor featuring an engineered tunnel barrier, known as the MAHONOS stack (gate metal/Al_2_O_3_/HfO_2_/SiO_2_/Si_3_N_4_/SiO_2_/Si), respectively. Figure 1c,d show the typical electrical characteristics of the transfer (I_D_-V_G_) and output (I_D_-V_D_) curves, respectively. In Figure 1c, the drain current (I_D_) is measured at a constant drain voltage (V_D_) of 100 mV while applying a gate voltage (V_G_) that ranges from −2 V to 4 V and then back to −2 V (V_G_ sweep rate, 50 mV/step) for a dual-sweep operation. The threshold voltage (V_th_) was extracted via linear extrapolation from the I_D_-V_G_ curve in the linear region. Additionally, the hysteresis voltage (ΔV_th_) was defined as the difference between the forward sweep (V_th_^f^) and backward sweep (V_th_^b^), calculated as ΔV_th_ = V_th_^f^ − V_th_^b^. In Figure 1d, I_D_ was measured as V_G_-V_th_ changes from 0 V to 4 V across 11 steps. The current linearly increased in the low V_D_ region and subsequently pinched off as V_D_ increased, resulting in saturation characteristics.

The electrical parameters of the SOI-based charge-trapping synaptic transistors were derived using the following equations [5,22]:

(1) $$SS={\left[\left(\frac{dlogI_{D}}{dV_{G}}\right)\right]}^{-1}$$

and

(2) $$\mu_{FE}=\left(\frac{Lg_{m}}{W\cdot C_{ox}\cdot V_{D}},g_{m}=\frac{\partial I_{D}}{\partial V_{G}}\right)$$

where *W*, *L*, *C_ox_*, and g*_m_* are the channel width, length, capacitance per unit area of the gate oxide, and transconductance, respectively. The extracted values for the V_th_, on/off current ratio (I_on_/I_off_), field-effect mobility (*μ*_FE_), and subthreshold swing were approximately −0.09 V, 9.35 × 10^7^, 209.87 cm^2^/V∙s, and 204.52 mV/dec, respectively. Table 1 provides a summary of the electrical parameters extracted from the SOI-based charge-trapping synaptic transistors.

**Figure 2:**
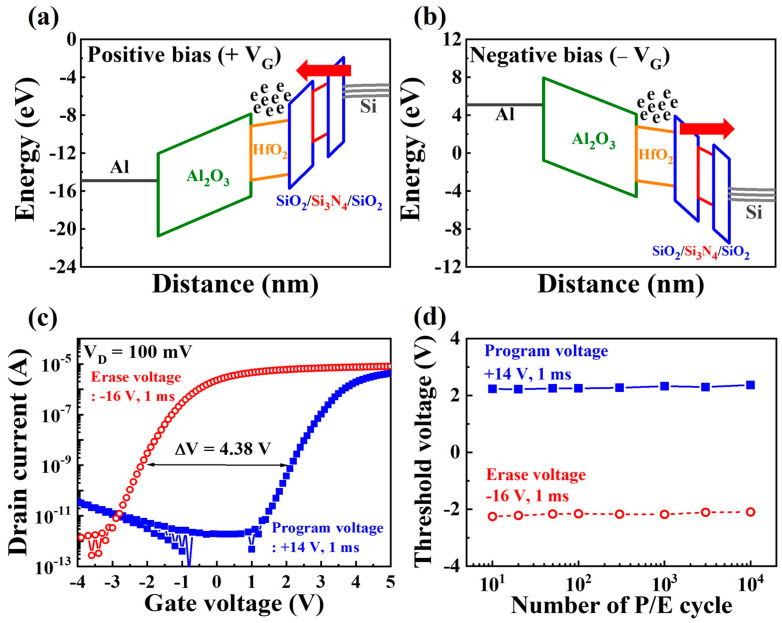
Energy band diagrams of the MAHONOS stack under (**a**) positive (V_G_ > 0 V) and (**b**) negative (V_G_ < 0 V) gate bias conditions. (**c**) Transfer curves and (**d**) endurance characteristics for erase and program states.

Figure 2a,b show the energy band diagrams of the MAHONOS stack under positive (V_G_ > 0 V) and negative (V_G_ < 0 V) gate bias conditions, respectively. In these diagrams, the Al_2_O_3_ layer serves as the BL, offering a high dielectric constant, a significantly large bandgap offset, and substantial physical oxide thickness (POT). The HfO_2_ layer was selected as the CTL because of its higher trap density, higher dielectric constant, and lower bandgap offset compared with the Al_2_O_3_ or SiO_2_ layers [23]. The engineered ONO structure, known as the VARIOT tunnel barrier, exhibits remarkable sensitivity to the electric field (E-field) generated by the gate bias [24]. When the energy of the electrons in the silicon channel is lower than that of the potential barrier, the thick ONO barrier prevents electron penetration. However, a substantial E-field causes significant band bending within the ONO barrier, allowing the electron wave function to tunnel through the thin triangular potential barrier [25]. Consequently, the channel conductance in the CTL can be modulated via charge trapping (resulting in a decrease in the conductance) or de-trapping (yielding an increase in the conductance). In retention mode, trapped charges remain stable in the CTL, and the charge-loss rate diminishes because of the substantial POT of the ONO barrier [26]. Figure 2c presents the transfer curve characteristics for the erase and program states. During programming, a gate bias of +14 V (for 1 ms) was applied, whereas erasing employed a bias of −16 V (for 1 ms). The threshold voltage shift attributed to charge trapping or de-trapping is approximately 4.38 V. Figure 2d shows the nonvolatile retention performance, demonstrating stable memory operation over 10^4^ s based on the program/erase cycle counts. All V_th_ values remained unaltered during the 10^4^-s observation period in both states.

### 3.2. Synaptic Characteristics of CMOS-Compatible Charge-Trapping Synaptic Transistors

The operation of synaptic transistors, which are the fundamental computing engines in the human brain, is pivotal for emulating biological synaptic functions and mechanisms. In a synaptic transistor system, an additional gate electrode serves as the presynaptic terminal, and the drain current (I_D_) simulates the EPSC. As signals traverse presynaptic terminals, EPSCs synchronize via a specially functionalized gate oxide layer, thereby emulating neural actions [27,28]. 

**Figure 3:**
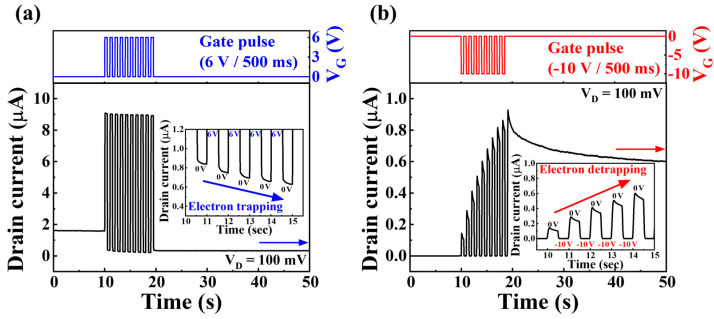
Gradual modulation of silicon channel conductance in I_D_ via (**a**) charge trapping (using positive V_G_ pulses) and (**b**) charge de-trapping (employing negative V_G_ pulses) in the charge-trapping layer.

Figure 3 shows the modulation of I_D_ via controlled charge-trapping and charge-de-trapping dynamics in the CTL via an engineered ONO structure with a VARIOT tunneling barrier. This modulation was achieved by sequentially applying gate pulses. Positive or negative gate pulses caused differences in the barrier height within the E-field-sensitive VARIOT tunneling barrier, leading to charge trapping or de-trapping in the CTL via Fowler–Nordheim tunneling. Consequently, the channel conductance was gradually modulated based on the charge quantity within the CTL. Figure 3a illustrates the gradual trapping of electrons within the CTL via the consecutive application of N = 10 positive gate pulses (6 V/500 ms). Subsequently, I_D_ sequentially decreased during the reading pulses at 0 V. Conversely, in Figure 3b, the application of 10 consecutive negative gate pulses (−10 V/500 ms) resulted in the gradual de-trapping of electrons within the CTL. Consequently, I_D_ sequentially increased during the reading pulses at 0 V.

**Figure 4:**
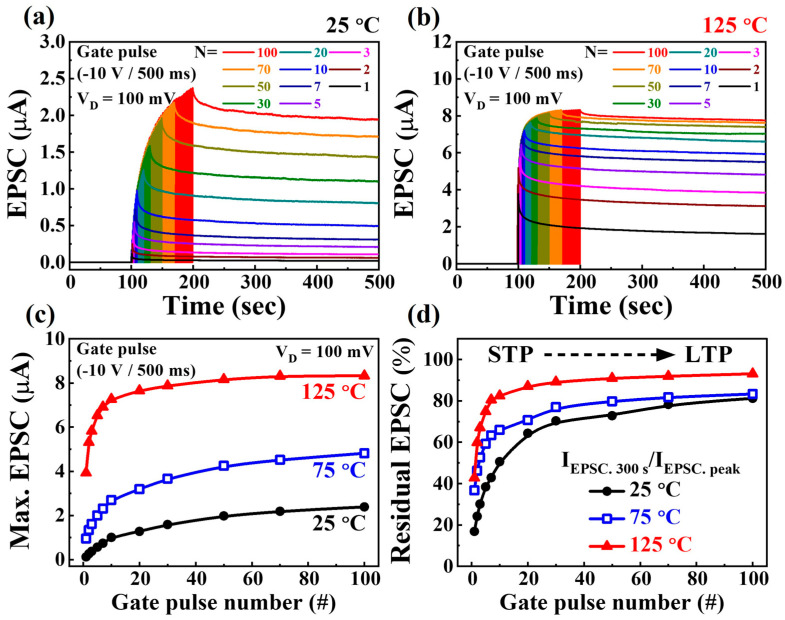
Excitatory-postsynaptic current (EPSC) responses in relation to gate pulse number (N = 1–100) at (**a**) 25 °C and (**b**) 125 °C. (**c**) Maximum EPSC values and (**d**) residual EPSC ratio as a function of gate pulse number across different temperatures (25, 75, and 125 °C).

Electronic devices capable of operating in challenging high-temperature environments are crucial for control, computing, communication, surveillance, and reconnaissance. At elevated temperatures, the intrinsic carrier concentration (n_i_) of semiconductors is nonnegligible, which adds significance to neuromorphic AI systems for rapid automation and intelligent decision-making [29,30,31]. To comprehensively assess the reliable conductance modulation and temperature dependency of SOI-based charge-trapping synaptic transistors, we employed multiple gate pulses (N = 1–100) and measured the EPSC response under varying temperature conditions (25, 75, and 125 °C). Figure 4a,b illustrate typical EPSC responses as a function of gate pulse number (N = 1–100) at 25 and 125 °C, respectively. As the gate pulse numbers increased, the trapped charges within the CTL progressively de-trapped, resulting in a gradual increase in the EPSC. Notably, the individual EPSC states corresponding to the pulse number were sustained over extended periods after the gate pulse stimuli. Figure 4c summarizes the maximum EPSC (I_EPSC-peak_) values immediately following gate pulse stimuli under varying temperature conditions (25, 75, and 125 °C). The magnitude of the I_EPSC-peak_ increased with an increase in the gate pulse number, showing further enhancement at higher temperatures. Particularly at elevated temperatures, even a few stimulus pulses yielded rapid EPSC escalation. This phenomenon occurs because the charges trapped within the CTL require less energy to tunnel via the VARIOT tunnel barrier under high-temperature conditions [32,33]. The conventional learning/memory mechanism proposed by Atkinson and Shiffrin for biological neural systems underscores the transition from STM to LTM via stimulus rehearsal. This transition is experimentally evident in synaptic transistors [34,35]. Figure 4d shows the transition from STM to LTM in charge-trapping synaptic transistors featuring an engineered tunnel barrier. This was achieved using the gate pulse number-dependent residual EPSC ratio (I_EPSC-300s_/I_EPSC-peak_), where I_EPSC-300s_ represents the resting EPSC value at 300 s post-gate stimulus completion. At 25 °C, the residual EPSC ratio was 16.9% after a single-gate pulse, indicating STM marked by swift EPSC decay. With increasing pulses, the ratio progressively increased, culminating in an LTM ratio of 81.2% after the 100th gate pulse. At 75 °C (or 125 °C), the residual EPSC ratios were 36.7% (or 42.8%) after a single stimulus and 83.3% (or 93.1%) after the 100th stimulus. In STM operation, the EPSC rapidly decreased post-peak owing to the Coulombic repulsion of charges within the CTL [36]. Conversely, the LTM operation exhibited a prolonged EPSC duration owing to the lower Coulombic repulsion upon complete de-trapping of charges in the CTL. Consequently, trapped charge modulation via gate pulse stimulation facilitates channel conductance transition from STM to LTM, which is viable at both low and high temperatures [37,38].

To gain more insight into the charge-trapping behavior within the CTL, the activation energy (E_a_) of the trap was derived from the temperature dependence between the I_EPSC_ and the number of gate pulse stimuli (N = 1–100) using the following Arrhenius equation:

(3) $$ln\left(I_{EPSC}\right)\propto \left({-E}_{a}/k_{B}T\right)$$

where *k_B_* and *T* represent the Boltzmann constant and the measured temperature (25, 75, and 125 °C, respectively) [39].

**Figure 5:**
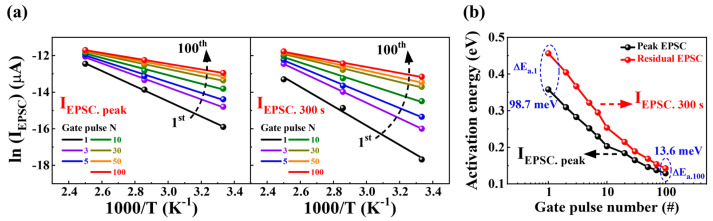
(**a**) Arrhenius plots depicting the relationship between I_EPSC-peak_ and I_EPSC-300s_ and the gate pulse number across the temperature range of 25–125 °C. (**b**) Gate pulse number-dependent activation energy (E_a_) for both I_EPSC-peak_ and I_EPSC-300s_.

Figure 5a illustrates the Arrhenius plots of both I_EPSC-peak_ and I_EPSC-300s_ against the gate pulse number across the temperature range of 25–125 °C. Figure 5b shows the variation in E_a_ for both the I_EPSC-peak_ and I_EPSC-300s_ corresponding to different gate pulse numbers. In I_EPSC-peak_, E_a_ transitioned from 0.36 eV (for a single-gate pulse, E_a-1_) to 0.13 eV (for the 100th gate pulse, E_a-100_), indicating a decrease with increasing pulse number. Similar trends were observed for the I_EPSC-300s_ domain, where E_a-1_ decreased from 0.46 eV to E_a-100_′s 0.14 eV as the number of gate pulses increased. The significant difference between E_a-1_ and E_a-100_ indicates that, initially, substantial band bending occurs because of the higher potential of the trapped charges within the CTL. Subsequent sequential gate pulse stimulations facilitate the de-trapping of charges, leading to a weakened potential of the less-trapped charges within the CTL. This de-trapping process consequently reduces the activation energy, contributing to the lowering of E_a_ values. Moreover, the difference in E_a_ between the I_EPSC-peak_ and I_EPSC-300s_ (ΔE_a_ = E_a-300s_ − E_a-peak_) provides insight into the number of trapped charges within the CTL. During the ΔE_a-1_ phase, the electrons induced in the silicon channel immediately after gate stimulation are hampered by the strong Coulombic repulsion of the charges trapped within the CTL. This dynamic reinforces the band-bending effect, significantly elevating ΔE_a-1_ post-pulse completion. Conversely, ΔE_a-100_ was significantly lower than ΔE_a-1_ due to the thorough de-trapping of charges within the CTL, indicating an LTM state characterized by reduced state changes over time.

The reinforcement of synaptic weight via repetitive stimuli signifies long-term changes, referred to as “long-term plasticity”, in contrast to “short-term plasticity.” Long-term potentiation and long-term depression represent the persistent strengthening and weakening of synaptic weights, respectively [40,41].

**Figure 6:**
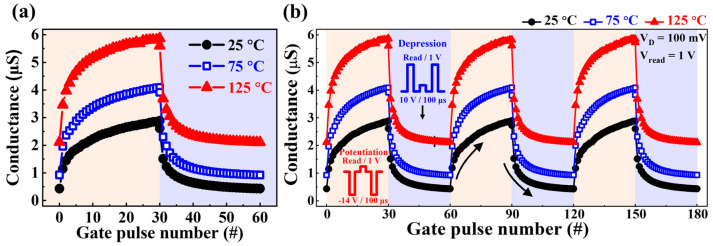
(**a**) Sequential channel conductance potentiation/depression behaviors induced by presynaptic pulses at temperatures of 25, 75, and 125 °C. (**b**) Endurance characteristics were demonstrated via repeated three-cycle cycles at temperatures of 25, 75, and 125 °C.

Figure 6a illustrates the sequential conductance potentiation and depression characteristics achieved by consecutive short gate pulse stimulation over three cycles of 100 μs each under temperature conditions of 25, 75, and 125 °C, respectively. Reliable modulation of channel conductance in individual synaptic transistors enables the realization of large-scale ANN systems via in-memory computing. Each conductance modulation cycle involves N = 30 potentiation pulses (−14 V/100 μs) followed by N = 30 depression pulses (10 V/100 μs). Depending on the sequence of potentiation and depression gate pulses, the channel conductance increased or decreased within the dynamic range (DR) of approximately 2.4, 3.2, and 3.7 μS at 25, 75, and 125 °C, respectively. Furthermore, as shown in Figure 6b, the channel conductance was consistently modulated during three repeated endurance cycles at both room and high temperatures. This remarkable weight variability at short stimulation times and elevated temperatures in the SOI-based charge-trapping synaptic transistors contributes to their versatility in applications as artificial synaptic devices. 

### 3.3. Modified National Institute of Standards and Technology Artificial Neural Network Recognition Simulation of Devices

Finally, a three-layer perceptron network model was proposed to simulate the learning of MNIST handwritten digits to validate the neuromorphic computing performance using the proposed synaptic devices. To design the ANN model, the normalized conductance and other parameters were initially calculated.

**Figure 7:**
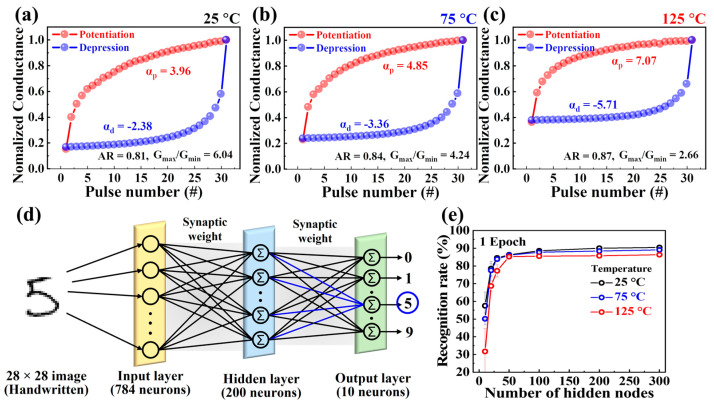
Normalized potentiation and depression (G_#_/G_max_) by nonlinearity at (**a**) 25, (**b**) 75, and (**c**) 125 °C of complementary metal-oxide semiconductor-compatible silicon-on-insulator-based charge-trapping synaptic transistors. (**d**) The architecture of a fully connected artificial neural network model comprising input, hidden, and output layers via synaptic weights for modified National Institute of Standards and Technology recognition simulation. (**e**) Recognition rates vary with the number of hidden neurons during epoch 1.

Figure 7a–c illustrate the normalized conductance for potentiation and depression in CMOS-compatible charge-trapping synaptic transistors at temperatures of 25, 75, and 125 °C, respectively. The normalized conductance (*G_#_*/*G_max_*) was obtained by calculating the ratio of conductivity at each step (*G_#_*) to the maximum conductivity (*G_max_*). These values were used as synaptic weights to represent the strength of the connections between neurons in the developed model for the recognition simulation. Examination of nonlinearity in normalized conductance allowed us to gain insights into crucial factors, such as the asymmetry ratio (AR), DR, and linearity, which greatly affect learning and recognition accuracy. The DR, defined as the ratio of *G_max_* to *G_min_*, represents the conductance modulation range, with higher values indicating improved performance and accuracy in the simulations [42]. The proposed CMOS-compatible SOI-based charge-trapping synaptic transistors exhibited DR values of 6.04 (25 °C), 4.24 (75 °C), and 2.66 (125 °C) at different temperatures, reflecting a decrease as the temperature increased between 25 °C and 125 °C. AR quantified the asymmetry between the potentiation and depression conductivities and was calculated using the following equation [43]:

(4) $$AR=\frac{MAX\left|G_{p}\left(n\right)-G_{d}\left(n\right)\right|}{G_{p}\left(30\right)-G_{d}\left(30\right)}\;for\;n=1\;to\;30$$

where *G_p_*(*n*) and *G_d_*(*n*) denote the conductivity values corresponding to the nth potentiation and depression pulses, respectively. An AR value close to 0 indicates optimal conditions with improved learning accuracy. The extracted AR values for the proposed device under different temperature conditions are 0.81 (25 °C), 0.84 (75 °C), and 0.87 (125 °C), respectively, indicating their proximity to the most ideal value at room temperature and symmetric conductivity changes.

To assess the linearity of the crucial conductance in the recognition simulations, the nonlinearity coefficient was defined using the following equation [44]:

(5) G={(Gmaxα−Gminα)×w+Gminα}1αGmin×(Gmax/Gmin)wif α≠0,if α=0.

where *G_max_* and *G_min_* represent the maximum and minimum conductivity values, respectively, and w ranges from 0 to 1. The ideal values for α_p_ and α_d_ are both 1, signifying the nonlinear factors controlling potentiation (α_p_) and depression (α_d_). The values of α_p_ and α_d_ extracted at different temperatures for the proposed CTF-type synaptic transistors were 3.96 and −2.38 (25 °C), 4.85 and −3.36 (75 °C), and 7.07 and −5.71 (125 °C), respectively, indicating higher linearity in conductivity modulation at relatively lower temperatures [45].

Subsequently, the obtained parameters and normalized conductance characteristics were used to design the ANN model. Figure 7d shows the fully connected synaptic weight network among the input, hidden, and output layers of the designed multilayer ANN model. The input layer consisted of 784 neurons connected to 28 × 28 pixels of the MNIST data, whereas the output layer comprised 10 neurons corresponding to digits 0–9. Each layer composed of multiple neurons represents an output value using a sigmoid activation function. The strength of the connections between the neurons in each layer was determined using the normalized conductance of the proposed CMOS-compatible SOI-based charge-trapping synaptic transistors. This model was used to assess the neuromorphic computing capability of the MNIST learning task. The ANN was trained using approximately 60,000 MNIST images from the training dataset for the simulation, and the recognition rate was evaluated by varying the number of hidden nodes from 10 to 300. Figure 7e depicts how the recognition rate changed with the number of hidden nodes during epoch 1, demonstrating an increase in the recognition rate as the number of hidden nodes increased. Notably, the recognition rates with 300 hidden nodes were 90.45% (25 °C), 89.07% (75 °C), and 86.3% (125 °C). The proposed CMOS-compatible SOI-based charge-trapping synaptic transistors exhibited relatively high recognition rates at room temperature, 75 °C, and 125 °C. This highlights the significant potential of the proposed device as a building block for AI applications and neuromorphic computing in high-temperature environments. 

## 4. Conclusions

We introduced CMOS-compatible SOI-based charge-trapping synaptic transistors featuring engineered tunnel barriers utilizing high-k dielectrics for applications in artificial synaptic electronics. A comprehensive evaluation of their essential electrical properties and artificial neural behaviors was systematically conducted. Tunnel barrier engineering realized using an ONO-stacked VARIOT structure with sequential HfO_2_ and Al_2_O_3_ stacking for the CTL and BL, respectively, yielded exceptional results. The fabricated MAHONOS-stacked synaptic transistors exhibit outstanding electrical characteristics. In the context of multiterminal synaptic behavior, we observed the modulation of silicon channel conductance via charge trapping and de-trapping at the CTL facilitated by gate pulse stimulation. Furthermore, the meticulously engineered tunnel barrier, which is responsive to gate pulses, enabled the reliable establishment of long-term potentiation and depression properties. The intrinsic variability of the silicon channel weights was demonstrated by the maximal EPSC aligned with the number of gate stimuli. This phenomenon signifies a transition from STM to LTM and is succinctly expressed by the residual EPSC ratio. Moreover, the charge trapping of the CTL was extensively elucidated by examining E_a_ according to the Arrhenius relationship between the I_EPSC_ and the corresponding measured temperatures. Additionally, the successive potentiation and depression of channel conductance, executed via short 100 μs gate stimuli, were consistently observed at both room and elevated temperatures, reaffirming their robustness. In conclusion, our learning simulations conducted on the MNIST handwritten digit dataset impressively demonstrate the capacity to achieve high recognition rates, even under high-temperature conditions. This underscores the viability of effectively emulating biological synapses. As a result, the proposed SOI-based charge-trapping synaptic transistor, specifically engineered with tunnel barriers, aligns seamlessly with CMOS processes and attests to exceptional versatility and reliability in in-memory computing for ANN applications, particularly when confronted with demanding environments characterized by elevated temperatures.

## Figures and Tables

**Table 1:** Electrical parameters of silicon-on-insulator-based charge-trapping synaptic transistors: threshold voltage (V_th_), on/off current ratio, field-effect mobility (*μ*_FE_), and subthreshold swing (*SS*).

Total Parameter			
V_th_ [V]	On/Off Current Ratio	µ_F_E [cm^2^/V×s]	SS [mV/dec]
−0.09	9.35 × 10^7^	209.87	204.52

## Data Availability

Not applicable.
